# “Catch me if you can” - locating the “Black Sheep” neurons after early-life seizures

**DOI:** 10.1186/s42494-024-00174-3

**Published:** 2024-10-25

**Authors:** Yingying Tang, Xiongfeng Guo, Mengqi Yan, Cenglin Xu

**Affiliations:** 1 Department of Pharmacy, Women’s Hospital, School of Medicine, Zhejiang University, Hangzhou, 310006 China; 2 Key Laboratory of Neuropharmacology and Translational Medicine of Zhejiang Province, School of Pharmaceutical Science, Zhejiang Chinese Medical University, Hangzhou, 310053 Zhejiang China

## Abstract

Unprovoked seizures in early life are one of the most severe conditions in pediatric neurology, and are often associated with long-lasting cognitive and behavioral deficits, as well as pharmacoresistant epilepsy in adulthood in some conditions. Unillustrated mechanisms greatly restrict the development of preventive strategies for early-life seizures (ELSs) related neuronal impairments. The recent groundbreaking study published in *The Journal of Clinical Investigation* represents a giant leap forward in understanding the complex pathogenesis mechanism and developing targeted therapies for ELS related neuronal impairments. The authors conducted elegant experiments to locate the activated pyramidal neuron subpopulation in the hippocampus and demonstrated the altered functions of (α-amino-3-hydroxy-5-methyl-4-isoxazole propionic acid)-type glutamate receptors (AMPARs). And we believe that the conclusions of this study may assist in further translational efforts to identify preventive targets for neurological disorders associated with early life seizures and propose new avenues for further exploration in this field.

## Background

Early-life unprovoked seizures are among the most severe diseases in pediatric neurology, frequently linked to persistent cognitive and behavioral deficits [1, 2], as well as in certain cases, pharmacoresistant epilepsy in adulthood [3]. The development of preventive therapies for neurological abnormalities associated with early-life seizures (ELSs) is significantly limited by the lack of demonstrated mechanisms. Cumulative evidences have suggested that abnormal excitatory neural networks and related cellular alterations (such as those in glial cells) may be involved in the pathological process after ELSs [4]. In which, recent trends have considered some distinct crucial neuronal subpopulations response heterogeneously in seizure activities [5]. It’s possible that those aberrantly seizure-activated neurons during the crucial stage of synaptogenesis are responsible for the long-lasting behavioral alterations.

## Main text

**Fig. 1:**
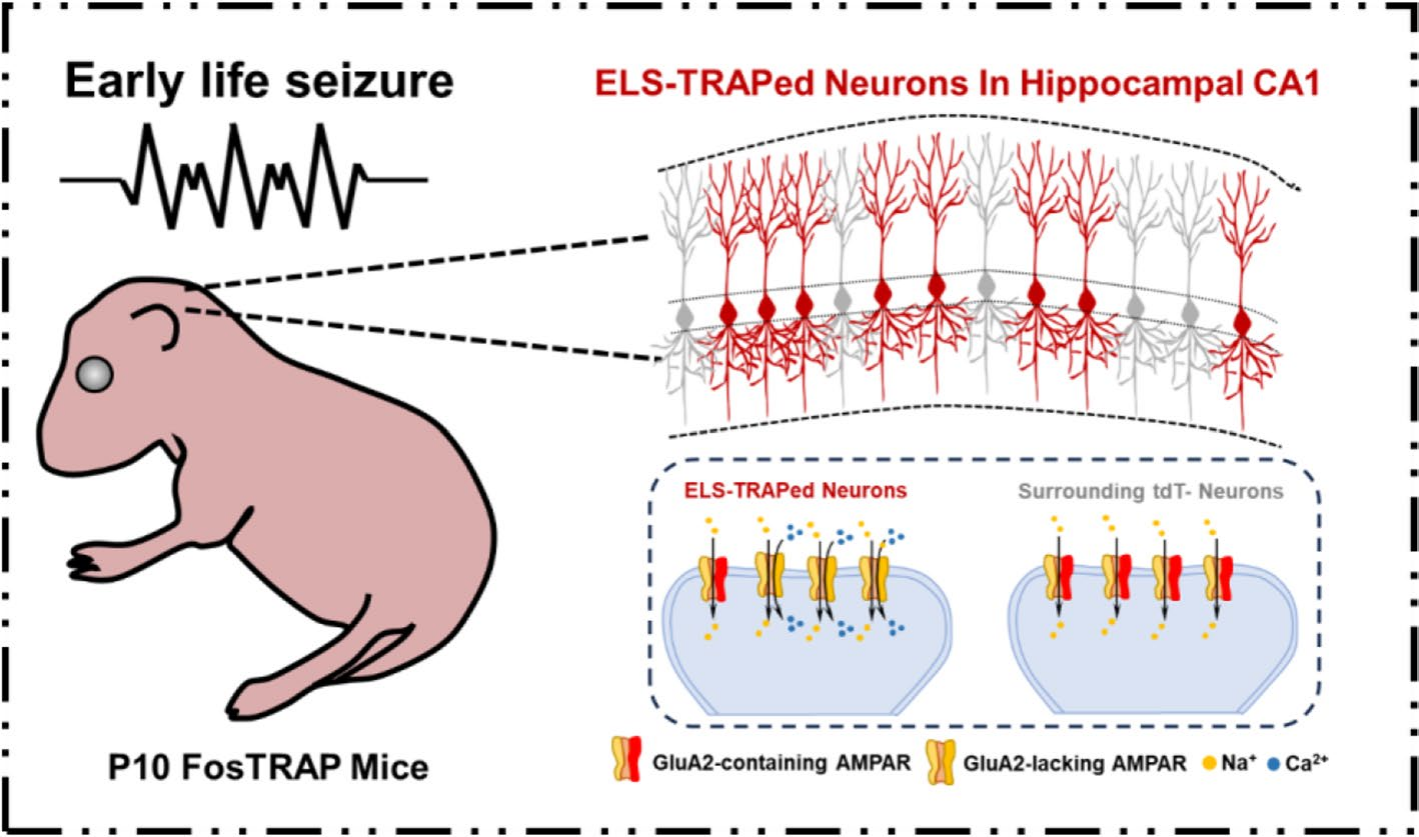
Early-life seizures activate a subpopulation of hippocampal pyramidal neurons and influence the functions of postsynaptic AMPA receptors (modified from the original article [6]). Fos TRAP mice were treated with KA to induce seizures at P10, leading to the activation of a subpopulation of pyramidal neurons in hippocampal CA1, which resulted in an increase in GluA2-lacking AMPARs in those ELS-TRAPed neurons

To investigate this theory, a recent elegant study published in *The Journal of Clinical Investigation* by a research group from the University of Pennsylvania has made significant efforts to identify the so-called “black sheep” neurons following ELS [6]. This groundbreaking work not only sheds light on the complex pathogenesis mechanisms of ELSs, but also pave a new way for developing targeted therapies for ELS related neuronal impairments (Fig. 1). The Fos targeted recombination in active population (FosTRAP) transgenic mice (FosTRAP mice) were used to reproduce the widely recognized kainic acid (KA)-induced seizure model at postnatal day 10 (P10). The addition of 4-hydroxytamoxifen, which stimulated the expression of tdTomato (tdT), allowed seizure-activated subpopulation cells to be persistently tagged following the tonic-clonic convulsions induced by KA. Through using this method, the authors claimed that at P28−P35, the KA-treated mice exhibited no overt cell death and had more ELS-TRAPed tdT^+^ cells than their saline-treated littermates. Further mapping the distribution of ELS-TRAPed cells revealed that the isocortex and hippocampus displayed the largest numbers of tdT^+^ cell, especially in the hippocampal CA1 region. It is noteworthy that there is a positive correlation between the number of ELS-TRAPed cells and the severity of seizures. Approximately 95% of the ELS-TRAPed cells were found to be pyramidal neurons. A second seizure assault was carried out on P10 KA-treated mice in order to determine whether those ELS-TRAPed pyramidal neurons would contribute to hyperexcitability and later-life seizures (LLSs). The authors reported that immunostaining performed after LLSs demonstrated a high degree of overlap between c-Fos positive cells and ELS-TRAPed pyramidal neurons in the CA1 region, and that mice with prior ELSs exhibited more severe seizure activity during the LLSs. Considering the whole picture, the authors came to the conclusion that ELS TRAPed neurons continued to be preferentially vulnerable to LLSs and might represent an epileptogenesis-related seizure engram.

The authors then made an effort to look into the molecular underpinnings of the contributions of ELS-TRAPed pyramidal neurons to hyperexcitation. Further in vitro electrophysiological recordings were conducted on ELS-TRAPed pyramidal neurons in this investigation. Although the intrinsic characteristics remained unchanged, AMPAR-mediated spontaneous excitatory postsynaptic currents (sEPSCs) showed greater amplitudes in tdT^+^ neurons compared to tdT^−^ neurons. Since GluA2-lacking, Ca^2+^-permeable AMPARs have been linked to persistent synaptic dysregulation after ELSs [7], the authors wanted to determine whether this behavior was specific to ELS-TRAPed pyramidal neurons. The tdT^+^ neurons displayed markedly increased rectification of AMPAR EPSCs through the detection of the inwardly rectifying currents, indicating an enlarged population of GluA2-lacking AMPARs. Consequently, the NMDA (N-methyl-D-aspartate)/ AMPA ratio was lower in ELS-TRAPed pyramidal neurons. These electrophysiological findings were further examined by RNAscope in situ hybridization, which revealed a significantly lower *Gria2/Gria1* ratio (encoding for GluA1 and GluA2, respectively) in the tdT^+^ neurons. Additionally, GluA2-synapsin colocalization was also decreased. This alteration was ascribed to the increased phosphorylation of GluA2 at Ser880, which therefore facilitated AMPAR endocytosis. In order to gain a better understanding of how ELS interfered with AMPARs during development, the scientists used protocols involving minimally induced EPSCs and failure rate to analyze silent synapses in ELS-TRAPed neurons. Interestingly, the proportion of silent synapses dramatically decreased in the tdT^+^ neurons after ELSs. Consequently, the inductions of both long-term potentiation (LTP) and long-term depression (LTD) were impaired in ELS-TRAPed neurons, while no changes were observed in the surrounding tdT^−^ neurons.

Lastly, the authors attempted to find a way to restore the synaptic functional deficit in the ELS-TRAPed neurons by consecutively injecting IEM-1460 (a selective blocker of GluA2-lacking AMPARs) after ELSs in FosTRAP mice [8]. The ELS-induced activation of pyramidal neurons in the CA1 region could not be affected by this therapy. On the other hand, in ELS-TRAPed neurons, it was able to effectively reverse the rectification of AMPAR-mediated currents. Additionally, the disruption of LTP and LTD in that neuron subset was repaired by post-seizure IEM-1460 therapy. The authors ultimately tested whether IEM-1460 also attenuated the ELS- induced disruption of LTP and LTD at the network level, and discovered that most slices taken from mice treated with IEM-1460 showed LTP, while fewer slices from mice treated with the vehicle showed LTP.

Taken together, this intriguing work provides direct evidence for the location of specific ELS-activated neurons in hippocampal CA1 and, for the first time, identifies the chemical underpinnings underlying the decreased synaptic activity in those neurons (Fig. 1). These fascinating findings expand our understanding of neurological diseases associated with ELS by identifying the specific cell subpopulation and highlighting GluA2-lacking AMPARs as possible therapeutic targets. Based on this, perhaps some future efforts could be paid towards both more in-depth mechanisms and translational significance. Firstly, although a transgenic mouse model was used, species differences still exist, posing challenges in translating these findings into clinical treatment strategies. Further preclinical and clinical researches are required to address these challenges. Perhaps replicating the crucial findings in samples obtained from ELS patients could establish sound foundations. Secondly, the current study primarily focuses on neuronal changes after ELS in a relative short period. Long-term follow-up studies to evaluate the impact of ELS on adulthood may be also important. The study has revealed the connection between changes in AMPA receptors and neuronal dysfunction following ELS, based on such crucial findings, the audience may wish to explore more in-depth mechanisms behind these changes. Moreover, since ELS-TRAPed neurons had defective post-synaptic AMPARs, scientists might also be interested in learning if this neuron subpopulation’s pre-synaptic input would be affected. Additionally, it would be intriguing to reconstruct the upstream and downstream circuits of ELS-TRAPed neurons using viral tracing to identify the pathogenic brain networks that follow ELS [9]. Furthermore, since ELSs could result in several neurological disorders, such as adult pharmacoresistant epilepsy, cognitive decline, and mood problems, it would be worthwhile to investigate if IEM-1460 or other comparable treatments targeting GluA2-lacking AMPARs could be effective in preventing these conditions. Further experiments are needed to validate its effects across different models and conditions, as well as to assess its safety and efficacy over the long term. By doing this, the necessary steps to open numerous clinical applications can be established.

## Conclusions

In conclusion, this study demonstrates that modifications of AMPARs occur in a subpopulation of ELS-activated hippocampal neurons, contributing to synaptic dysplasticity and network hyperexcitability caused by ELS. It also presents that GluA2-lacking AMPARs as potential targets for preventing adulthood pharmacoresistant epilepsy or related neurological conditions caused by ELS.

## Data Availability

Not applicable.

## Abbreviations

AMPAR: (α-amino-3-hydroxy-5-methyl-4-isoxazole propionic acid)-type glutamate receptor
ELS: Early-life seizure
FosTRAP: Fos targeted recombination in active populations
KA: Kainic acid
tdT: tdTomato
LLS: Later-life seizure
LTP: Long-term potentiation
LTD: Long-term depression
sEPSCs: Spontaneous excitatory postsynaptic currents
